# Detection of Zika virus in paired urine and amniotic fluid samples from symptomatic and asymptomatic women and their babies during a disease outbreak: association with neurological symptoms in newborns

**DOI:** 10.1007/s13365-019-00797-0

**Published:** 2019-09-09

**Authors:** Danila Vedovello, Steven S. Witkin, Andrea Cristina Botelho Silva, Thamirys Cosmo Gillo Fajardo, Alexandra Siqueira Mello, Ana Paula Antunes Pascalicchio Bertozzi, Alify Bertoldo da Silva, Nemésio Florence Vieira Filho, Maria Manoela Duarte Rodrigues, Rosa Estela Gazeta, Antônio Fernandes Moron, Stéphanno Gomes Pereira Sarmento, Antonio Soriano-Arandes, Saulo Duarte Passos

**Affiliations:** 1 Laboratory of Pediatric Infectology, Jundiaí School of Medicine, Rua Francisco Telles, 250, Vila Arens, Jundiaí, SP 13202-550 Brazil; 2 Jundiaí School of Medicine, University Hospital, Jundiaí, SP Brazil; 3 Weill Cornell Medicine, New York, NY USA; 4 Neonatal Perinatal Medical Forum, Paulista Federal Medical School, São Paulo, SP Brazil; 5 Paulista Center for Fetal Medicine, Paulista Federal Medical School, São Paulo, SP Brazil; 6 Pediatric Infectious Diseases Unit, Hospital Universitari Vall d’Hebron, Barcelona, Spain

**Keywords:** Zika virus, Pregnancy, Neurological symptoms, Urine, Amniotic fluid

## Abstract

Paired maternal and newborn urine and amniotic fluid from 138 subjects collected during a Zika virus (ZIKV) outbreak was analyzed for ZIKV by gene amplification (RT-qPCR), and the findings were correlated with clinical symptoms and neurological anomalies in the babies. ZIKV was detected in 1 of 9 symptomatic women (11.1%) and in 19 of 129 asymptomatic women (14.7%). Neurological manifestations were present in 19 babies (13.7%), 10 of 20 (50%) positive and 9 of 119 (7.6%) negative (*p* < 0.001) for ZIKV. Twelve (8.6%) urines collected during gestation were ZIKV-positive; only 2 remained positive for ZIKV postpartum. Six (4.1%) newborn urines collected within 1 day of delivery were ZIKV-positive cases. In 3 of these cases, ZIKV was detected in mother’s urine pre- and postpartum and in both mother’s urine and babies’ urine. Four of the amniotic fluid samples (2.9%) were ZIKV-positive. Among ZIKV-negative babies with neurological sequel, 87.5% were female; in contrast, 72.7% ZIKV-positive babies with neurological abnormalities were male (*p* = 0.019). We conclude that during a ZIKV outbreak, clinical symptoms and ZIKV detection in biological fluids are poor predictors of infection and adverse neurologic sequel in newborns.

## Introduction

Zika virus (ZIKV) is an arbovirus and member of the *Flaviviridae* family that also includes the Yellow Fever Virus, Dengue Virus, West Nile Virus, Japanese Encephalitis Virus, St. Louis Encephalitis Virus, and others (Simmonds et al. 2017). ZIKV (strain MR-766) was initially isolated in the Uganda forest from sentinel monkeys in 1947 (Dick et al. 1952). It has been claimed that the first description of ZIKV in humans occurred in Nigeria in 1954 (MacNamara 1954). However, studies have shown that this may have been confused with the presence of the Spondweni virus (Simpson et al. 1964; Haddow and Woodall 2016), another Flavivirus that produces symptoms similar to ZIKV and is serologically cross-reactive. Therefore, the first description of ZIKV in humans is now attributed to Simpson and collaborators in 1964 (Simpson et al. 1964).

ZIKV re-emerged in 2007, with an epidemic on Yap Island, Micronesia (Duffy et al. 2009) followed by its spread to Oceania and to the Americas (Musso and Gubler 2016). The first detection of autochthonous ZIKV in Brazil occurred in 2015. (Zanluca et al. 2015). Initial phylogenetic analyses of seven ZIKV sequences suggested its introduction into Brazil, from endemic areas of Oceania (Faria et al. 2016). However, subsequent studies have shown that these Brazilian strains were also closely related to ZIKV strains isolated in Haiti, Central America, and the Caribbean Islands, suggesting several potential routes of spread (Campos et al. 2018).

According to the World Health Organization (WHO), the case definition of a symptomatic ZIKV infection includes a rash and/or fever and at least one of the following: arthralgia, arthritis, or conjunctivitis (WHO 2016). However, the range of symptoms varied in different affected regions. In French Polynesian and the Americas, outbreaks were generally mild and accompanied by fever, arthralgia and myalgia, conjunctivitis, headache, fatigue, and/or rash. In other regions, ZIKV elicited more severe symptoms in adults, including multi-organ failure, meningitis, encephalitis, and thrombocytopenia (Pierson and Diamond 2018).

ZIKV virulence for pregnant women and their babies in Brazil first became apparent in May 2015 (Cugola et al. 2016). The ZIKV replication cycle begins with transmission from an infected mosquito (Weaver and Reisen 2010). After initial replication in epidermal keratinocytes and Langerhans cells, the virus migrates to lymph nodes in association with monocytes, macrophages, and dendritic cells (Jurado and Iwasaki 2017). Following a primary viremia, ZIKV may infect cells in multiple organs such as the spleen, kidney, and testes. It may also cross the blood-brain barrier. In pregnant women, ZIKV can infect placental macrophages (Quicke et al. 2016).

ZIKV has been detected in blood (Jurado and Iwasaki 2017), saliva (Barzon et al. 2016), urine (Gourinat et al. 2015; Zhang et al. 2016), fetal brain (Oliveira Melo et al. 2016), and semen (Mansuy et al. 2016; Counotte et al. 2018), in cases of renal transplantation (Nogueira et al. 2017) and in amniotic fluid (Calvet et al. 2016; Benjamin et al. 2017).

In the present study, we investigated associations between maternal symptom occurrence, ZIKV detection in maternal or newborn urine and amniotic fluid, and the presence of neurological symptoms in male and female babies.

## Material and methods

### Subjects

The study population consisted of 138 mother-infant pairs identified from an ongoing cohort of high-risk pregnant women during a ZIKV outbreak in the city of Jundiai, State of São Paulo, Brazil, in 2016–2017. A subject was defined as having a high-risk pregnancy if she had a history of miscarriage or preterm birth or was positive for gestational diabetes mellitus (GDM), arterial hypertension, history of toxoplasmosis, hepatitis C, urinary tract infection, syphilis or other sexually transmitted diseases, an autoimmune disorder, or being obese. The inclusion criterion was the availability of maternal urine samples collected both during gestation and after delivery, amniotic fluid collected at the time of delivery, and newborn urine collected within 1 day of birth. All infants were followed up for a period of 1 year.

The presence of symptoms consistent with criteria established by the WHO for possible ZIKV infection was ascertained by direct questioning. Subjects’ race was as self-reported.

All subjects provided written informed consent authorizing the use of de-identified biological samples from themselves and their babies for subsequent scientific investigation. The study was approved by the Research Ethics Committee of the Jundiai School of Medicine, CAAE 53248616.2.0000.5412.

### Sample collection

Urine samples obtained from mothers during their gestation and after delivery and from their babies within 1 day of birth were collected in sterile pouches. Amniotic fluid was collected with a 20-mL syringe at the time of delivery by an obstetrician at the University Hospital by a technique that we developed. If the delivery was by cesarean section, the obstetrician induced membrane rupture with tweezers and inserted a syringe to collect 5–10 mL of amniotic fluid. In cases of vaginal delivery, the obstetrician induced rupture of the amniotic membrane with tweezers or waited for spontaneous rupture and then collected 5–10 mL of amniotic fluid from the vagina. All samples were transferred to a sterile tube, placed in ice-water, and sent immediately to the Laboratory of Pediatric Infectious Diseases for analysis. The amniotic fluid was centrifuged at 1400*g* for 20 min at 4 °C and the supernatant aliquoted into vials and stored at − 80 °C. Nine of the 138 births were twins. However, only one of these twin pregnancies was dizygotic yielding a total of 139 amniotic fluid samples for analysis.

### ZIKV detection

All urine and amniotic fluid samples were analyzed for ZIKV by quantitative reverse transcriptase–polymerase chain reaction (RT-qPCR) as recommended by the Centers for Disease Control and Prevention (CDC) (Lanciotti et al. 2008). We recognize that PCR positivity may be restricted to a variable period after infection and findings should ideally be interpreted in regard to time since exposure. This was not possible in our endemic setting. The RT-qPCR was performed using the GoTaq® 1-Step RT-qPCR System (© 2018 Promega, Madison, WI, USA).

RNA was extracted from the biological samples by the QIAamp Viral RNA Kit (© 2018, Qiagen, Venlo, Netherlands), following the manufacturer’s instructions. Afterwards, 8 μL of RNA template was mixed with 10 μL of GoTaq® Probe qPCR Master Mix with dUTP (1×) with 10% of Carboxy-X-Rhodamine (CXR), 0.4 μL of GoScript™ RT Mix for 1-Step RT-qPCR (1×), 1 μL of forward primer (10 pmol/μL), 1 μL of reverse primer (10 pmol/μL), 1 μL of probe (10 pmol/μL), and nuclease-free water to complete the final volume of 25 μL. Two sets of primers were used, serving as a double check.

Amplification was performed on an ABI Prism 7500 SDS Real-Time cycler Applied Biosystems (© 2016 Thermo Fisher Scientific, Waltham, MA, USA) and consisted of a 10-min cycle at 50 °C and a 2-min cycle at 95 °C to produce cDNA; and forty cycles of 15 s at 95 °C and 1 min at 60 °C for the PCR (Lanciotti et al. 2008). Three positive controls (RNA extracted from ZIKV-positive samples) and three negative controls, nuclease-free H_2_O (Thermo Fisher Scientific, Waltham, MA, USA) were included in each run. Samples that were amplified by the two sets of primers were considered positive. In all cases, the positive and negative controls yielded the expected results.

### Statistics

Associations between numerical variables were analyzed by Fisher’s exact test. Means and standard deviations were obtained using the Mann-Whitney test (Mann and Whitney 1946). A two-sided *p* value < 0.05 was considered significant.

## Results

Characteristics of the study population are shown in Table 1. Mean age of the mothers was 28.8 years, mean gestational age at delivery was 37.9 weeks, and mean baby birth weight was 3113 g. Nine of the women (6.5%) delivered twins. The majority of the deliveries were by cesarean section (82.7%) and most of the women were either White (44.6%) or of mixed race (31.6%).

**Table 1:** Characteristics of the study population

Characteristic	Value
Mothers age (years)	28.8 (6.8)^a^
Gestational age at delivery (weeks)	37.9 (2.2)^a^
Babies birthweight (g)	3113 (643)^a^
Twins	9 (6.5%)
Delivery by cesarean section	82.7%
Male baby	44.6%
Female baby	55.4%
White	44.6%
Mixed race	31.6%
Black	10.8%
Asian	0.7%

The study population consisted of 138 mother-baby pairs

^a^Mean (standard deviation)

Of the 138 urine samples collected from pregnant mothers before delivery, 12 (8.6%) were ZIKV-positive. Postpartum, only 2 of the previously positive urine samples remained positive for ZIKV. Urine collected within 1 day of delivery from the 148 newborns (139 singletons and 9 twins) was ZIKV-positive in 6 (4.1%) cases. ZIKV was detected in 4 (2.9%) of the 139 amniotic fluid samples collected from the 138 women. In the dizygotic twin gestation, ZIKV was detected in one of the amniotic sacs. In total, ZIKV was detected in one of 9 women (11.1%) who were positive for ZIKV-associated symptoms and in 19 of 129 women (14.7%) who were asymptomatic.

The diagnoses of microcephaly and macrocephaly in our cohort were made using the INTERGROWTH-21(st) parameters (Papageorghiou et al. 2013). Neurological manifestations were detected in 15 babies at the time of delivery (9 cases of microcephaly 5 cases of macrocephaly 1 case of macrocraniania) and in an addition, four babies only at later time periods. These four cases included (a) dystonia (297 days after birth), cataract, and abnormal respiratory system in the newborn of an asymptomatic mother who was positive for ZIKV only in urine while pregnant; (b) aggravating cranial developmental problems (254 days after birth) and low visual acuity in the newborn of an asymptomatic mother, positive for ZIKV only in in urine while pregnant; (c) ventricular system of abnormal dimensions (parallelism of the lateral ventricles) and ventricular system of abnormal morphology (discrete duct distension of the supratentorial system) in the newborn of a symptomatic mother positive for ZIKV only in urine while pregnant; (d) ataxia (in 11th month of life), binocular low visual acuity (in 5th month of life), abnormal swallowing, and respiratory tract abnormal in a newborn positive for ZIKV in its urine.

Neurological manifestations were identified in babies born in 10 of 20 women (50.0%) who were ZIKV-positive vs. only 9 of 119 women (7.6%) who were ZIKV-negative (*p* < 0.001). Results for each of the mother-baby pair positive for ZIKV and/or neurological symptoms are shown in Table 2. In nine cases where both mother and baby were ZIKV-negative, 8 asymptomatic and one symptomatic mother, the baby manifested a neurological abnormality at birth—5 cases of microcephaly and 4 cases of macrocephaly. Of nine cases where only the mother’s urine was positive during pregnancy, 4 of their babies were apparently normal, 2 had microcephaly, 1 had macrocephaly, and 2 had late sequela. One case where both pregnancy and postpartum urine were ZIKV-positive was associated with a baby with macrocrania. When both pregnancy and postpartum urine and newborn urine were ZIKV-positive, the baby had microcephaly. In the six cases where only the newborns’ urine was positive, two of the babies did not manifest any abnormalities, 2 had microcephaly, and 2 had late neurological manifestations. In the 4 cases of ZIKV detection only in amniotic fluid, all babies appeared unaffected. Among the ZIKV-positive mothers, 3 entered the high-risk pool due to GDM and one each because of obesity, hypothyroidism, chronic arterial hypertension, pulmonary disease, or a hematological disorder. The distribution of risks necessitating entry into the high-risk pregnancy pool was not different between ZIKV-positive or -negative mothers and there was no association between newborn neurological manifestations and identified pregnancy risk (data not shown). Strikingly, among the ZIKV-negative babies with neurological sequel, 87.5% were female; conversely, 72.7% of ZIKV-positive babies with neurological abnormalities were male (*p* = 0.019).

**Table 2:** Symptoms, Zika virus detection, and neurological problems in the newborn

Symptoms	ZIKV in				Disorder
	Pregnancy urine	Postpartum urine	Newborn urine	Amniotic fluid	
Asymptomatic					
1	Negative	Negative	Negative	Negative	Macrocephaly
2	Negative	Negative	Negative	Negative	Microcephaly
3	Negative	Negative	Negative	Negative	Macrocephaly
4	Negative	Negative	Negative	Negative	T1 microcephaly
					T2 negative
5	Negative	Negative	Negative	Negative	Microcephaly
6	Negative	Negative	Negative	Negative	Microcephaly
7	Negative	Negative	Negative	Negative	Microcephaly
8	Negative	Negative	Negative	Negative	T1 negative
					T2 macrocephaly
9	Positive	Negative	Negative	Negative	Negative
10	Positive	Negative	Negative	Negative	Negative
11	Positive	Negative	Negative	Negative	Negative
12	Positive	Negative	Negative	Negative	Negative
13	Positive	Negative	Negative	Negative	Dystonia, cataracts, ventricle defects
14	Positive	Negative	Negative	Negative	Microcephaly
15	Positive	Negative	Negative	Negative	Macrocephaly
16	Positive	Negative	Negative	Negative	Microcephaly
17	Positive	Positive	Negative	Negative	Macrocraniania
18	Positive	Positive	Positive	Negative	Microcephaly
19	Negative	Negative	Negative	Positive	Negative
20	Negative	Negative	Negative	Positive	Negative
21	Negative	Negative	Negative	T1 positive	Negative
				*T2* negative	Negative
22	Positive	Negative	Negative	Positive	Negative
23	Negative	Negative	Positive	Negative	Macrocephaly, duct distension
24	Negative	Negative	Positive	Negative	Ataxia, vision, and respiratory problem
25	Negative	Negative	Positive	Negative	Negative
26	Negative	Negative	Positive	Negative	Negative
27	Negative	Negative	Positive	Negative	Microcephaly
Symptomatic					
1	Negative	Negative	Negative	Negative	Macrocephaly
2	Positive	Negative	Negative	Negative	Ventricular abnormalities, duct distension

T1/T2, twin gestations

*116 newborns were negative for ZIKV and did not present at birth or late sequelae. So as their mothers, they were not positive for ZIKV either

## Discussion

Consequences of ZIKV in pregnant women are variable among different populations and factors associated with the most serious pathology—neurological disorders in their newborns—remain incompletely determined. In the present study of a high-risk pregnancy population evaluated during the time of a ZIKV outbreak, we report that adverse neurological sequela was identified in 50% of cases in which women had ZIKV detected in their urine during gestation and/or postpartum and/or in their newborn’s urine. Thus, these findings are consistent with previous studies on the strong association between ZIKV infection during pregnancy and neurological pathology in their offspring (Walter et al. 2018; Russo et al. 2017; Li et al. 2016) and, furthermore, that maternal ZIKV infection does not always adversely affect the involved neonates (Pomar et al. 2018). Newborn neurological problems were also identified in 7.6% of cases in which ZIKV was not detected in any sample. This implies that, especially during ZIKV outbreaks, the absence of ZIKV detection does not necessarily mean that the fetus will not become infected and suffer adverse consequences.

All babies with adverse neurological disorders were from mothers with ZIKV-negative amniotic fluid and none of the 4 cases of ZIKV detection in amniotic fluid was associated with a fetal neurological anomaly.

Babies with neurological defects but who along with their mothers were negative for ZIKV detection were almost all female, while, conversely, neurological defects in association with maternal or neonatal ZIKV detection were almost all in males. This suggests a possible influence of fetal sex on ZIKV titer at different body sites and/or variations in the likelihood of transplacental infection. These areas deserve further study.

Our observations were made during a ZIKV outbreak. This suggests the involvement of this virus in the neurological sequela, even in those cases where ZIKV was not detected by RT-qPCR. ZIKV-positive urine is a transient occurrence (Zhang et al. 2016) and may have been no longer present in some of the positive cases. Our findings complement previous investigations highlighting the variable consequences for newborns of having a mother who is ZIKV-positive (Wheeler et al. 2018) as well as the occurrence of adverse neurological consequences in babies whose mothers did not have detectable ZIKV during their gestation (Nogueira et al. 2018). The rates of detection of ZIKV in maternal or neonatal urine in our study population is consistent with what has been reported previously (Gourinat et al. 2015) and is most likely a consequence of the transient nature of ZIKV infection at this site (Zhang et al. 2016). In addition, it is possible that ZIKV may not infect the urinary tract in all cases of infection.

Limitations of the present study need to be acknowledged. Symptoms in our pregnant population were self-reported and so are prone to recall bias. We attempted to minimize this possibility by conducting interviews with highly trained personnel, but this remains a possibility. The timing of ZIKV infection has been shown to influence the rate of viral detection in biological fluids, the occurrence of transplacental viral passage, and neonatal adverse effects (Cao et al. 2017). Unfortunately, in the present study, this information could not be obtained. Almost all subjects came to our clinic in their late second trimester. The lack of concordance between ZIKV detection at the different sampling sites in most subjects in our study might possibly be due to technical failure, although our assay for ZIKV detection included controls that ruled out the possible presence of inhibitors of gene amplification. Differences in viral load between samples at the various sites and/or variations in ZIKV concentration at the same site between individual women would also account for the observed inconsistencies in viral detection. These samples were collected post-delivery. Conversely, we acknowledge that our technique of collecting amniotic fluid from women with vaginal delivery is prone to contamination by maternal blood and/or vaginal secretions.

There are multiple infectious and genetic causes of microcephaly and macrocephaly (Devakumar et al. 2018; Gilmore and Walsh 2012; Passemard et al. 2013). Unfortunately, we were unable to test for antibodies to *flaviviruses*. There is a hypothesis that the simultaneous presence of more than one flavivirus infection may potentiate negative sequelae. However, these studies are not definitive and need further exploration (Badolato-Correa et al. 2018). Even in animal models, the results are still inclusive (Langerak et al. 2019).

Other arboviruses such as Dengue and Chikungunya are common in Brazil. Therefore, the contribution of other viruses to the observed neurological problems cannot be definitely excluded. Macrocephaly has been previously been described in ZIKV-positive neonates (Levine et al. 2017; Chimelli et al. 2017), and the present study supports this as a possible ZIKV-related outcome. One of the babies with macrocephaly was from a mother with GDM. In this case, we cannot distinguish if this neurological problem was a consequence of the GDM, ZIKV infection, or both. Further investigations are required to determine under which circumstances a congenital ZIKV infection can predispose to macrocephaly.

We conclude that while ZIKV detection in maternal and newborn urine is associated with the subsequent occurrence of neurological problems in newborns or at later times in postnatal development, the variables associated with transplacental passage of the virus and induction of neurological pathology in the developing baby still remain largely undetermined. In addition, due at least in part to individual variability in rates of viral replication and/or persistence at different sites, the absence of ZIKV detection does not necessarily mean the absence or infection or guarantee that the fetus was not infected or may have neurological abnormalities. Therefore, we strongly recommend that periodic ultrasound measurements are performed in all fetuses regardless of the mothers’ ZIKV status during periods of ZIKV prevalence.
